# (*E*)-Methyl 3-(3,4-dihy­droxy­phen­yl)acrylate

**DOI:** 10.1107/S1600536810054504

**Published:** 2011-01-12

**Authors:** Li Wang, Fa-Yan Meng, Cui-Wu Lin, Hai-Yan Chen, Xuan Luo

**Affiliations:** a College of Chemistry and Chemical Engineering, Guangxi University, Guangxi 530004, People’s Republic of China

## Abstract

The benzene ring in the title compound, C_10_H_10_O_4_, makes an angle of 4.4 (1)° with the C—C—C—O linker. The hy­droxy groups are involved in both intra- and inter­molecular O—H⋯O hydrogen bonds. The crystal packing is stabilized by O—H⋯O hydrogen-bonding inter­actions. The mol­ecules of the caffeic acid ester form a dimeric structure in a head-to-head manner along the *a* axis through O—H⋯O hydrogen bonds. The dimers inter­act with one another through O—H⋯O hydrogen bonds, forming supermolecular chains. These chains are further extended through C—H⋯O hydrogen bonds as well as van der Waals inter­actions into the final three-dimensional architecture.

## Related literature

For properties of caffeic acids and their esters, see: Altug *et al.* (2008 ▶); Ates *et al.* (2006 ▶); Atik *et al.* (2006 ▶); Chun *et al.* (2008 ▶); Huang *et al.* (2010 ▶); Hwang *et al.* (2001 ▶); Padinchare *et al.* (2001 ▶). For a polymorphic form of the title compound, see: Chen *et al.* (1979 ▶).
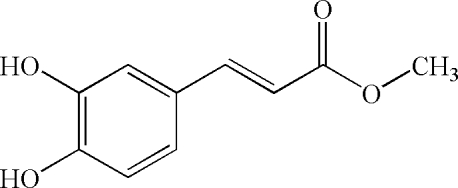


## Experimental

### 

#### Crystal data

C_10_H_10_O_4_
*M* *_r_* = 194.18
Triclinic, 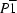

*a* = 5.129 (5) Å
*b* = 9.969 (8) Å
*c* = 10.586 (9) Å
α = 117.627 (7)°
β = 97.924 (11)°
γ = 94.322 (11)°
*V* = 468.9 (7) Å^3^
*Z* = 2
Mo *K*α radiation
μ = 0.11 mm^−1^
*T* = 296 K
0.33 × 0.24 × 0.18 mm

#### Data collection

Multiwire proportional diffractometer
Absorption correction: multi-scan (*SADABS*; Bruker, 2005 ▶) *T*_min_ = 0.966, *T*_max_ = 0.981
2494 measured reflections
1619 independent reflections
1337 reflections with *I* > 2σ(*I*)
*R*_int_ = 0.013

#### Refinement

*R*[*F* ^2^ > 2σ(*F* ^2^)] = 0.040
*wR*(*F* ^2^) = 0.120
*S* = 1.05
1619 reflections
128 parameters
H-atom parameters constrained
Δρ_max_ = 0.18 e Å^−3^
Δρ_min_ = −0.13 e Å^−3^

### 

Data collection: *SMART* (Bruker, 2005 ▶); cell refinement: *SAINT* (Bruker, 2005 ▶); data reduction: *SAINT*; program(s) used to solve structure: *SHELXS97* (Sheldrick, 2008 ▶); program(s) used to refine structure: *SHELXL97* (Sheldrick, 2008 ▶); molecular graphics: *SHELXTL* (Sheldrick, 2008 ▶); software used to prepare material for publication: *SHELXTL*.

## Supplementary Material

Crystal structure: contains datablocks I, global. DOI: 10.1107/S1600536810054504/bg2376sup1.cif

Structure factors: contains datablocks I. DOI: 10.1107/S1600536810054504/bg2376Isup2.hkl

Additional supplementary materials:  crystallographic information; 3D view; checkCIF report

## Figures and Tables

**Table 1:** Hydrogen-bond geometry (Å, °)

*D*—H⋯*A*	*D*—H	H⋯*A*	*D*⋯*A*	*D*—H⋯*A*
O4—H4*A*⋯O3	0.82	2.28	2.723 (2)	114
O4—H4*A*⋯O3^i^	0.82	2.15	2.835 (2)	141
O3—H3*A*⋯O2^ii^	0.82	1.95	2.764 (2)	175
C10—H10*A*⋯O2^ii^	0.93	2.56	3.260 (4)	132

Symmetry codes: (i) 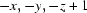; (ii) 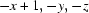.

**Fig. 1.**
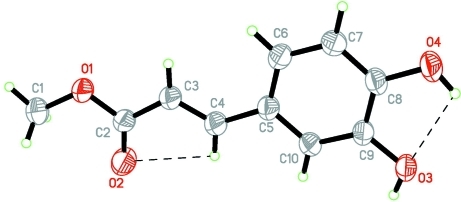
View of the molecular structure of (E)-methyl 3-(3,4-dihydroxyphenyl)acrylate with displacement ellipsoids at a 45% probability level. Dashed lines represent intramolecular hydrogen bonds.

**Fig. 2.**
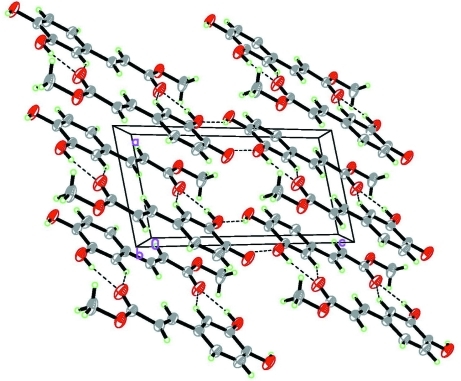
Packing of the molecules drawn along the b axis. Dashed lines represent hydrogen bonds.

## supplementary crystallographic information

### Comment

Some naturally occurring caffeic acids and their esters attract much attention in biology and medicine (Hwang *et al.*, 2001; Altug *et al.*, 2008). These compounds show antiviral, antibacterial, vasoactive, antiatherogenic, antiproliferative, antioxidant and antiinflammatory properties (Atik *et al.*, 2006; Padinchare *et al.*, 2001; Ates *et al.*, 2006). Our previous research found that the phenolic acids compounds including caffeic acid, chlorogenic acid, ferulic acid, vanillic acid, syringic acid and protocatechuic acid from Blumea riparia DC have a significant role at antiplatelet activity (Huang *et al.*., 2010). This prompted us to synthesize a series of caffeic acid esters and amides to investigate their properties. In this paper, we report a polymorph of C_10_H_10_O_4_ (Chen *et al.*., 1979).

In the title compound (Fig. 1), all values of the geometric parameters are normal. The benzene ring is planar within experimental error and it makes an angle of 4.4 (1)° to the linker (C2–C3–C4–O2). Hydroxy groups contribute to intermolecular O—H···O hydrogen bonds. In the case of caffeic esters, the presence of an ethylenic spacer allows the formation of a conjugated system, strongly stabilized through π-electron delocalization (Chun *et al.*, 2008).

The bond C3=C4 is a *trans*-double bond. The crystal packing is stabilized by intramolecular (Table 1, entries 1 and 2) and intermolecular hydrogen-bonding interactions (Table 1, remaining entries). The molecules of the caffeic acid ester form a dimeric structure through O—H···O hydrogen bonds along the *a* axis in a head-to-head manner (Table 1, third entry) . The dimer interacts with another dimmer through O—H···O hydrogen bonds (Table 1, fourth entry) to form one-dimensional supermolecule chains. These one-dimensional supermolecule chains are further extended through C—H···O hydrogen bonds *(*Table 1, fifth entry) as well as van der Waals interactions into the final 3-D architecture (Fig.2).

### Experimental

Commercial caffeic acid (1.79 g, 10 mmol) was dissolved in tetrahydrofuran solution(16 ml), followed by methanol (16 ml) and the addition of concentrated hydrochloric acid (8 ml). The mixture was stirred at 60 °C for 60 minutes, followed by the addition of water and extracted with ethyl acetate. The organic layer was washed with sodium bicarbonate and water, dried over magnesium sulfate, and concentrated to give a solid residue. The residue was recrystallized from petroleum ether to give the title compound as a colourless crystal (1.64 g, yield: 84%)

### Refinement

All the H atoms were positioned geometrically (C—H = 0.93–0.96 Å) and refined as riding with *U*_iso_ = 1.2Ueq.

### Crystal data

**Table:**

C_10_H_10_O_4_	*Z* = 2
*M**_r_* = 194.18	*F*(000) = 204
Triclinic, *P*1	*D*_x_ = 1.375 Mg m^−^^3^
Hall symbol: -P 1	Mo *K*α radiation, λ = 0.71073 Å
*a* = 5.129 (5) Å	Cell parameters from 1619 reflections
*b* = 9.969 (8) Å	θ = 2.2–25.0°
*c* = 10.586 (9) Å	µ = 0.11 mm^−^^1^
α = 117.627 (7)°	*T* = 296 K
β = 97.924 (11)°	Block, colourless
γ = 94.322 (11)°	0.33 × 0.24 × 0.18 mm
*V* = 468.9 (7) Å^3^	

### Data collection

**Table:**

Multiwire proportional diffractometer	1619 independent reflections
Radiation source: fine-focus sealed tube	1337 reflections with *I* > 2σ(*I*)
graphite	*R*_int_ = 0.013
phi and ω scans	θ_max_ = 25.0°, θ_min_ = 2.2°
Absorption correction: multi-scan (*SADABS*; Bruker, 2005)	*h* = −6→5
*T*_min_ = 0.966, *T*_max_ = 0.981	*k* = −11→11
2494 measured reflections	*l* = −12→11

### Refinement

**Table:**

Refinement on *F*^2^	Secondary atom site location: difference Fourier map
Least-squares matrix: full	Hydrogen site location: inferred from neighbouring sites
*R*[*F*^2^ > 2σ(*F*^2^)] = 0.040	H-atom parameters constrained
*wR*(*F*^2^) = 0.120	*w* = 1/[σ^2^(*F*_o_^2^) + (0.0615*P*)^2^ + 0.0896*P*] where *P* = (*F*_o_^2^ + 2*F*_c_^2^)/3
*S* = 1.05	(Δ/σ)_max_ < 0.001
1619 reflections	Δρ_max_ = 0.18 e Å^−^^3^
128 parameters	Δρ_min_ = −0.13 e Å^−^^3^
0 restraints	Extinction correction: *SHELXL97* (Sheldrick, 2008), Fc^*^=kFc[1+0.001xFc^2^λ^3^/sin(2θ)]^-1/4^
Primary atom site location: structure-invariant direct methods	Extinction coefficient: 0.023 (7)

### Special details

**Table:**

Geometry. All e.s.d.'s (except the e.s.d. in the dihedral angle between two l.s. planes) are estimated using the full covariance matrix. The cell e.s.d.'s are taken into account individually in the estimation of e.s.d.'s in distances, angles and torsion angles; correlations between e.s.d.'s in cell parameters are only used when they are defined by crystal symmetry. An approximate (isotropic) treatment of cell e.s.d.'s is used for estimating e.s.d.'s involving l.s. planes.
Refinement. Refinement of *F*^2^ against ALL reflections. The weighted *R*-factor *wR* and goodness of fit *S* are based on *F*^2^, conventional *R*-factors *R* are based on *F*, with *F* set to zero for negative *F*^2^. The threshold expression of *F*^2^ > σ(*F*^2^) is used only for calculating *R*-factors(gt) *etc*. and is not relevant to the choice of reflections for refinement. *R*-factors based on *F*^2^ are statistically about twice as large as those based on *F*, and *R*- factors based on ALL data will be even larger.

### Fractional atomic coordinates and isotropic or equivalent isotropic displacement parameters (Å^2^)

**Table:**

	*x*	*y*	*z*	*U*_iso_*/*U*_eq_	
O1	0.3038 (3)	0.37534 (14)	−0.18804 (13)	0.0565 (4)	
O2	0.5270 (3)	0.19675 (16)	−0.17913 (15)	0.0718 (5)	
O3	0.1400 (2)	−0.02223 (14)	0.35179 (13)	0.0548 (4)	
H3A	0.2389	−0.0773	0.3036	0.082*	
O4	−0.2041 (3)	0.17229 (17)	0.47736 (15)	0.0696 (5)	
H4A	−0.1537	0.1033	0.4914	0.104*	
C1	0.4334 (5)	0.3652 (2)	−0.3048 (2)	0.0689 (6)	
H1A	0.3752	0.4367	−0.3354	0.103*	
H1B	0.3878	0.2631	−0.3853	0.103*	
H1C	0.6232	0.3891	−0.2711	0.103*	
C2	0.3676 (3)	0.28238 (18)	−0.13522 (17)	0.0436 (4)	
C3	0.2244 (3)	0.29584 (18)	−0.02085 (17)	0.0445 (4)	
H3B	0.1053	0.3654	0.0075	0.053*	
C4	0.2628 (3)	0.20962 (19)	0.04297 (18)	0.0460 (4)	
H4B	0.3849	0.1428	0.0100	0.055*	
C5	0.1401 (3)	0.20498 (18)	0.15770 (17)	0.0424 (4)	
C6	−0.0350 (3)	0.30272 (19)	0.22626 (19)	0.0513 (5)	
H6A	−0.0774	0.3767	0.2001	0.062*	
C7	−0.1459 (4)	0.2904 (2)	0.3326 (2)	0.0568 (5)	
H7A	−0.2618	0.3568	0.3778	0.068*	
C8	−0.0874 (3)	0.1811 (2)	0.37298 (18)	0.0476 (4)	
C9	0.0889 (3)	0.08337 (18)	0.30649 (16)	0.0423 (4)	
C10	0.2006 (3)	0.09637 (18)	0.20077 (17)	0.0442 (4)	
H10A	0.3192	0.0312	0.1571	0.053*	

### Atomic displacement parameters (Å^2^)

**Table:**

	*U*^11^	*U*^22^	*U*^33^	*U*^12^	*U*^13^	*U*^23^
O1	0.0832 (9)	0.0569 (7)	0.0553 (7)	0.0308 (6)	0.0364 (6)	0.0392 (6)
O2	0.0865 (10)	0.0896 (10)	0.0850 (10)	0.0535 (8)	0.0538 (8)	0.0637 (8)
O3	0.0709 (8)	0.0646 (8)	0.0599 (7)	0.0330 (6)	0.0364 (6)	0.0458 (6)
O4	0.0859 (10)	0.0920 (10)	0.0775 (9)	0.0488 (8)	0.0561 (8)	0.0633 (8)
C1	0.1062 (17)	0.0689 (12)	0.0585 (11)	0.0311 (11)	0.0432 (11)	0.0430 (10)
C2	0.0502 (10)	0.0428 (8)	0.0462 (9)	0.0123 (7)	0.0160 (7)	0.0257 (7)
C3	0.0501 (10)	0.0474 (9)	0.0464 (9)	0.0156 (7)	0.0185 (7)	0.0275 (7)
C4	0.0511 (10)	0.0498 (9)	0.0487 (9)	0.0161 (7)	0.0205 (8)	0.0291 (8)
C5	0.0451 (9)	0.0463 (9)	0.0438 (8)	0.0104 (7)	0.0143 (7)	0.0263 (7)
C6	0.0596 (11)	0.0559 (10)	0.0588 (10)	0.0233 (8)	0.0246 (8)	0.0388 (9)
C7	0.0644 (12)	0.0650 (11)	0.0655 (11)	0.0341 (9)	0.0361 (9)	0.0418 (10)
C8	0.0520 (10)	0.0588 (10)	0.0467 (9)	0.0178 (8)	0.0233 (7)	0.0324 (8)
C9	0.0470 (9)	0.0475 (9)	0.0424 (8)	0.0121 (7)	0.0142 (7)	0.0278 (7)
C10	0.0490 (10)	0.0486 (9)	0.0456 (9)	0.0173 (7)	0.0209 (7)	0.0268 (7)

### Geometric parameters (Å, °)

**Table:**

O1—C2	1.325 (2)	C3—H3B	0.9300
O1—C1	1.449 (2)	C4—C5	1.460 (2)
O2—C2	1.206 (2)	C4—H4B	0.9300
O3—C9	1.372 (2)	C5—C6	1.392 (2)
O3—H3A	0.8200	C5—C10	1.395 (2)
O4—C8	1.361 (2)	C6—C7	1.378 (2)
O4—H4A	0.8200	C6—H6A	0.9300
C1—H1A	0.9600	C7—C8	1.381 (2)
C1—H1B	0.9600	C7—H7A	0.9300
C1—H1C	0.9600	C8—C9	1.391 (2)
C2—C3	1.460 (2)	C9—C10	1.378 (2)
C3—C4	1.327 (2)	C10—H10A	0.9300
			
C2—O1—C1	115.65 (14)	C6—C5—C10	118.11 (15)
C9—O3—H3A	109.5	C6—C5—C4	123.59 (15)
C8—O4—H4A	109.5	C10—C5—C4	118.30 (14)
O1—C1—H1A	109.5	C7—C6—C5	120.39 (15)
O1—C1—H1B	109.5	C7—C6—H6A	119.8
H1A—C1—H1B	109.5	C5—C6—H6A	119.8
O1—C1—H1C	109.5	C6—C7—C8	121.02 (16)
H1A—C1—H1C	109.5	C6—C7—H7A	119.5
H1B—C1—H1C	109.5	C8—C7—H7A	119.5
O2—C2—O1	122.42 (15)	O4—C8—C7	118.86 (15)
O2—C2—C3	124.94 (14)	O4—C8—C9	121.79 (15)
O1—C2—C3	112.64 (14)	C7—C8—C9	119.35 (15)
C4—C3—C2	120.52 (15)	O3—C9—C10	123.84 (14)
C4—C3—H3B	119.7	O3—C9—C8	116.62 (14)
C2—C3—H3B	119.7	C10—C9—C8	119.54 (14)
C3—C4—C5	129.05 (16)	C9—C10—C5	121.59 (14)
C3—C4—H4B	115.5	C9—C10—H10A	119.2
C5—C4—H4B	115.5	C5—C10—H10A	119.2
			
C1—O1—C2—O2	1.4 (3)	C6—C7—C8—O4	−179.27 (17)
C1—O1—C2—C3	−178.44 (15)	C6—C7—C8—C9	0.9 (3)
O2—C2—C3—C4	−0.5 (3)	O4—C8—C9—O3	−0.2 (2)
O1—C2—C3—C4	179.32 (15)	C7—C8—C9—O3	179.66 (16)
C2—C3—C4—C5	−179.72 (15)	O4—C8—C9—C10	179.64 (16)
C3—C4—C5—C6	−3.9 (3)	C7—C8—C9—C10	−0.5 (3)
C3—C4—C5—C10	175.86 (16)	O3—C9—C10—C5	179.43 (14)
C10—C5—C6—C7	−0.6 (3)	C8—C9—C10—C5	−0.4 (3)
C4—C5—C6—C7	179.19 (17)	C6—C5—C10—C9	0.9 (2)
C5—C6—C7—C8	−0.3 (3)	C4—C5—C10—C9	−178.84 (15)

### Hydrogen-bond geometry (Å, °)

**Table:**

*D*—H···*A*	*D*—H	H···*A*	*D*···*A*	*D*—H···*A*
O4—H4A···O3	0.82	2.28	2.723 (2)	114.
C4—H4B···O2	0.93	2.48	2.829 (4)	102
O4—H4A···O3^i^	0.82	2.15	2.835 (2)	141.
O3—H3A···O2^ii^	0.82	1.95	2.764 (2)	175.
C10—H10A···O2^ii^	0.93	2.56	3.260 (4)	132

Symmetry codes: (i) −*x*, −*y*, −*z*+1; (ii) −*x*+1, −*y*, −*z*.
